# Reinforcement Effect of CaCl_2_ on Cementation Performance of Solid-Waste-Based Cementitious Materials for Fine Tailings

**DOI:** 10.3390/molecules30071520

**Published:** 2025-03-29

**Authors:** Qing Liu, Yanan Wu

**Affiliations:** 1 School of Chemical Engineering and Environment, Weifang University of Science and Technology, Weifang 262700, China; 2 Shandong Provincial Key Laboratory of Water Pollution Control and Resource Reuse, School of Environmental Science and Engineering, Shandong University, Qingdao 266237, China; 201920359@mail.sdu.edu.cn

**Keywords:** fine tailings, cementing performance, acceleration effect, hydration products

## Abstract

Cemented paste backfill with mine tailings provides a desirable solution for maximally utilizing mine tailings. Ordinary Portland cement (OPC) is the most widely used binder for cemented tailings backfills; however, the serious environmental problems resulting from OPC production and the drawbacks of OPC in cementing fine tailings motivate the investigation of novel binders characterized by environmental friendliness, cost-effectiveness, and efficiency. We previously synthesized solid-waste-based cementitious materials (SWCMs) for cementing fine tailings. In this study, CaCl_2_ was added as an accelerator to further enhance the cementing performance of SWCMs for fine tailings. Adding a small amount of CaCl_2_ accelerated the hydration of raw materials and prompted the formation of larger amounts of hydration products. As a result, the cementing performance of SWCMs for fine tailings was significantly enhanced through the combined effect of C-S-H gel and ettringite. The cemented fine tailings backfill can be hardened only after curing for ~36 h, with a 50% decrease in hardening duration compared to the control sample without CaCl_2_. The optimal amount of CaCl_2_ was controlled at 1.5 wt.%, and the sample strength reached 0.21 MPa at 36 h, even at a low binder-to-tailings ratio of 1:8, meeting the requirement of early strength of common cemented tailings backfills. The rapid hardening of cemented fine tailings backfills has significant implications for accelerating ore mining speed, improving mining production capacity, ensuring the safe environment of underground mining sites, and preventing the movement of surface masses in the terrain where mining production takes place.

## 1. Introduction

With the increasing demand for mineral resources and the continuous progress of mineral processing technology, the mining output of ores characterized by low-grade and complex components is increasing year by year. As a result, the amount of mine tailings (MTs) discharged from the mineral processing has increased dramatically, and MT particles have become increasingly finer. The cumulative emissions of MTs in China have exceeded 15 billion tons alone between 2011 and 2020 [1,2,3]. The majority of MTs are piled up on the surface in the form of tailings ponds. Such a simple and rough disposal of MTs not only occupies large areas of land, which in many cases is also agricultural land, and pollutes the environment, but also presents a serious safety hazard [4]. Considering these adverse effects, it is imperative to reduce tailings emissions and improve the comprehensive utilization of tailings.

Recently, numerous relevant studies have been carried out, and some approaches for MT utilization, such as the re-separation of MTs, utilization of MTs as building materials, and utilization of MTs as a mine backfill material, have been presented. Cemented tailings backfill for underground mine goaf may radically alter the role of MTs in mines. The advantages of this technology can be summarized as follows [5,6,7]: (1) recycling clean water to save resources; (2) maximizing the utilization of tailings and reducing their storage in tailings ponds; (3) minimizing the environmental problems and safety hazards; (4) improving ore recovery rate and reducing ore dilution; (5) improving the stability of underground goaf and achieving safe and efficient mining of ores. This technology can promote the safe disposal of tailings, reduce surface subsidence, and provide ground support, which cannot be solved by other tailings treatment methods or can be achieved solely by high operating costs. Binders are an important component of cemented tailings backfills, and their cost can account for approximately 75% of the total cost of underground backfilling in mines [6]. In addition, the characteristics of binders are critical for the performance of cemented tailings backfills. Therefore, the research and development of binders for cementing tailings has attracted increasing attention.

Ordinary Portland cement (OPC) is the most widely used binder for cemented tailings backfills, with satisfactory performance for coarse tailings. However, the drawbacks of OPC in cementing fine tailings (in which the proportion of particles <20 μm exceeds 60%) have become increasingly prominent, such as large OPC consumption, high cost, low early strength, and high bleeding ratio [8,9,10]. Compared with OPC, alkali-activated cementitious materials (AACMs), which are prepared from solid wastes such as slag, fly ash, and industrial byproduct gypsum, possess excellent potential for cementing fine tailings [11,12]. Wang et al. [13] prepared cementitious materials with 76.92% slag, 19.24% carbide slag, and admixtures of 2.88% sodium silicate and 0.96% calcium chloride for cementing fine tailings, and it was found that the 3-day and 28-day compressive strength of the fine tailings cemented paste backfill with AACMs increased by 124% and 14%, respectively, compared to fine tailings cemented paste backfill with 42.5 OPC. Hu et al. [14] synthesized cemented tailings backfill with slag and fly ash as primary constituents and sodium silicate and NaOH as the alkaline activator, and the optimum compressive strength of cemented tailings backfill reached 4.4 MPa at 28 days. Li et al. [15] reported that the 28-day compressive strength of cemented ultrafine tailings backfill with steel slag, fly ash, and slag as the precursor and carbide slag and desulfurization gypsum as the activator could reach over 2.0 MPa, showing a significant increase of 26.70%~39.20% compared to that with OPC as the binder.

During our past works, solid-waste-based cementitious materials (SWCMs) from 42.5 wt.% slag, 27.5 wt.% steel slag (SS), 15.0 wt.% desulfurization gypsum (DG), and 15.0 wt.% OPC were synthesized. It was found that the 3-day and 28-day compressive strengths of SWCM cemented fine tailings backfills were above 0.2 and 1.0 MPa, respectively, reaching the strength requirement of common cemented tailings backfills [5,16,17,18]. In actual backfill engineering, the rapid hardening of cemented tailings backfills can not only accelerate ore mining speed and improve mining production capacity but also, more importantly, ensure the safe environment of underground mining sites and prevent the movement of surface masses in the terrain where mining production takes place [5,16]. Therefore, targeted approaches are necessary to further enhance the cementing performance of SWCMs for fine tailings. Accelerators are a water-soluble chemical additive, and a minor addition of them can significantly promote the hydration process, accelerate reactant formation, and thus improve the cementing performance of cementitious materials [19,20,21]. The effects of accelerators on OPC hydration have been widely studied; however, to our knowledge, the role of accelerators in the hydration process of multi-component composite SWCMs has rarely been reported, particularly when SWCMs are used as a binder for cementing fine tailings.

In this study, SWCMs served as a binder for cementing fine tailings, and anhydrous calcium chloride (CaCl_2_) as an accelerator was expected to prompt the hydration process of SWCMs and thus accelerate the hardening speed of cemented fine tailings backfills. The compressive strength, pH of the pore solution, hydration products, hydration heats, microstructural morphology, and pore structure of samples were analyzed to explore the mechanism of action of CaCl_2_ in the hydration process and reveal the cementing mechanism of SWCMs for fine tailings.

## 2. Results and Discussion

### 2.1. Effect of CaCl_2_ Content on Cementing Performance of SWCMs for Fine Tailings

In actual backfill engineering, the required strength may vary greatly depending on the expected function and the underground environment. Related studies have shown that the early strength (usually referring to 3-day strength) of the backfill material should be between 0.15 and 0.30 MPa to ensure its stability and provide support for workers and trackless equipment entering the goaf underground in mines [5,16]. In addition, the 28-day strength should not be lower than 1.0 MPa to meet the strength requirements of the underground support wall in the mine and ensure the safe environment of the mining site [17,18].

**Figure 1:**
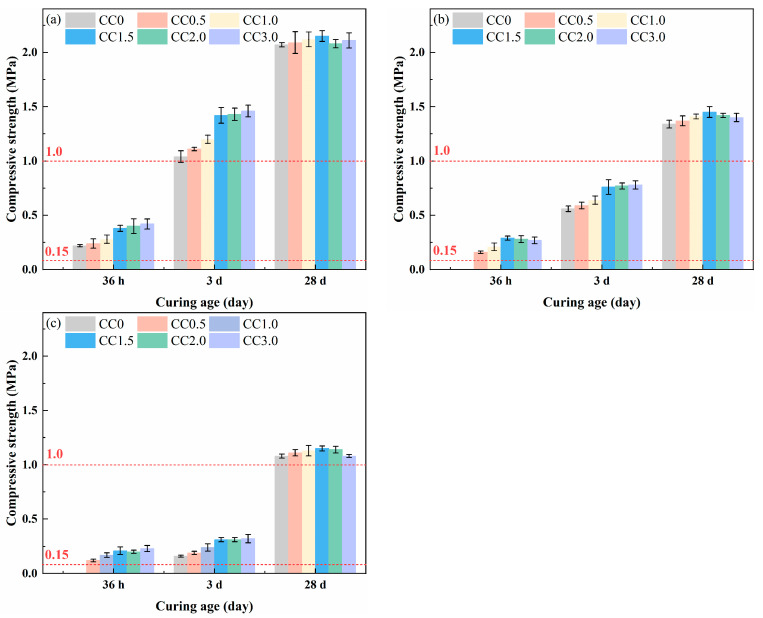
Compressive strength of cemented fine tailings backfill with different CaCl_2_ contents at the binder-to-tailings ratio of (**a**) 1:4, (**b**) 1:6, and (**c**) 1:8.

The compressive strengths of cemented fine tailings backfills are presented in Figure 1. The two red dotted lines represent the minimum strength of cemented fine tailings backfills at 3 days (0.15 MPa) and 28 days (1.0 MPa), respectively. It can be seen that the addition of CaCl_2_ increases the sample strength, especially during the early ages. Overall, with the increase in CaCl_2_ content, the compressive strength increased initially and then decreased slightly, and the optimum amount of CaCl_2_ was kept at 1.5 wt.%. For cemented fine tailings backfills at a binder-to-tailings ratio of 1:4, the samples could harden in only 36 h, and the strength continuously increased with the increase of CaCl_2_ content. In detail, the compressive strengths of samples CC0–CC3.0 were 0.22, 0.24, 0.28, 0.38, 0.40, and 0.42 MPa, respectively. This strength meets the requirement of early strength of cemented tailings backfills. As the curing time was prolonged to 28 days, the strength of samples CC0–CC3.0 increased to 2.07, 2.09, 2.12, 2.15, 2.08, and 2.11, respectively. Unlike the strength development at early ages, the sample strength reduced slightly when the CaCl_2_ content exceeded 1.5 wt.%.

The sample strength decreased with decreasing binder-to-tailings ratio because of the reduction in hydration reactants derived from the hydration of cementitious materials. Sample CC0 required 3 days to harden at the binder-to-tailings ratio of 1:6 or 1:8. The addition of CaCl_2_ enhanced the cementing performance of SWCMs for the fine tailings, and the cemented fine tailings backfills with CaCl_2_ can still harden after curing for 36 h. The compressive strength of sample CC1.5 is 0.29 and 0.21 MPa at the binder-to-tailings ratio of 1:6 and 1:8, respectively, reaching the strength requirement of cemented tailings backfills at 3 days. Shortened hardening time is of great significance for the practical application of cemented fine tailings backfills. During the process of underground backfill in mines, the fast hardening of cemented fine tailings backfills can not only accelerate the mining and filling speed, shorten the time for trackless equipment to enter the mining site, and improve the production capacity of the mine, but also ensure the safe environment of the underground mining site [5,16]. After curing for 28 days, the strength of sample CC1.5 increased to 1.40 and 1.08 MPa at the binder-to-tailings ratio of 1:6 and 1:8, respectively, meeting the 28-day strength requirement for underground backfill materials in mines (>1.0 MPa).

### 2.2. Effect of CaCl_2_ on the Hydration Properties of SWCMs

#### 2.2.1. pH of the Pore Solution of SWCMs

**Figure 2:**
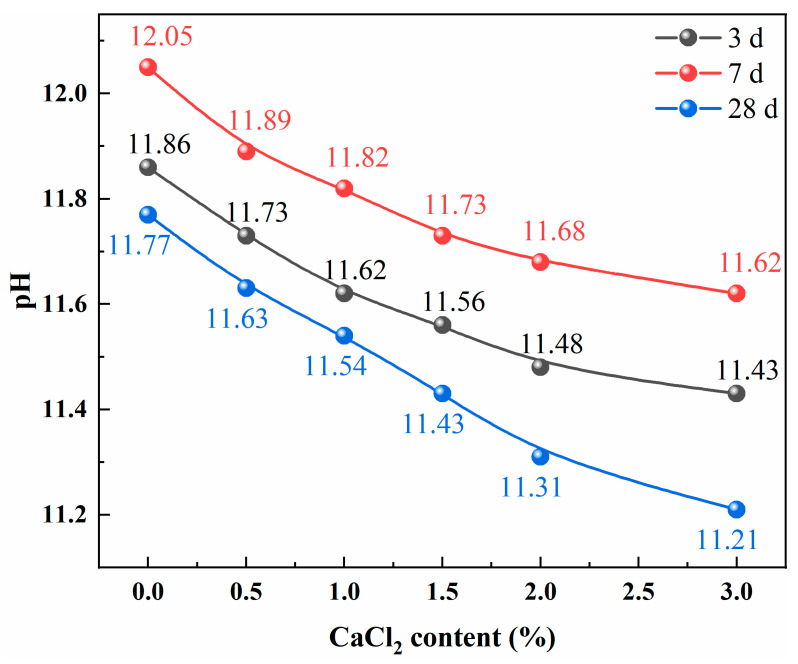
pH evolution of the pore solution of SWCMs under different CaCl_2_ contents.

The pH of the pore solution is known to play a crucial role in the dissolution and hydration of raw materials. It has been shown that the hydration reaction of cementitious materials can only proceed when the pH of the pore solution exceeds a certain threshold [22,23]. The evolution of the pH of the pore solution of SWCMs under different CaCl_2_ contents is shown in Figure 2.

The pH of the pore solution of hydrated samples continuously decreased with increasing CaCl_2_ content during the entire curing period. The pH of sample CC0 is the highest, with pH values of 11.86, 12.05, and 11.77 at 3, 7, and 28 days, respectively. After the CaCl_2_ content was increased to 3.0 wt.%, the pH of sample CC3.0 rapidly decreased to 11.43, 11.62, and 11.20 within the same curing age. For the SWCM system, the alkalinity of the pore solution is associated with the ionization of the Ca(OH)_2_ phase, which mainly comes from the hydration of C_2_S and C_3_S in cement and steel slag, as shown in reaction Equations (1)–(3) [24,25]. Therefore, the decrease in pH may be due to two reasons: (1) CaCl_2_ inhibits the hydration of C_2_S and C_3_S in cement and steel slag, thereby reducing the generation of Ca(OH)_2_. (2) The Ca(OH)_2_ phase generated by the hydration of C_2_S and C_3_S reacts with CaCl_2_ and transforms into other stable reactants. As can be seen from Figure 2, the addition of CaCl_2_ enhances the strength of SWCMs, which indicates that the hydration of cement and steel slag is not inhibited by CaCl_2_. As a consequence, the reduced pH most likely results from the second hypothesis. Literature reports suggest that CaCl_2_ can combine with Ca(OH)_2_ in cementitious materials to form insoluble double salts, as shown in reaction Equations (4) and (5) [26,27]. These phases with a large amount of bound water are stable and increase the proportion of the solid phase in the hydrated samples, facilitating the formation of a denser microstructure and improving the compressive strength of cementitious materials.

(1) $$3\mathrm{CaO}\cdot {\mathrm{SiO}}_{2}+xH_{2}O\to x\mathrm{CaO}\cdot {\mathrm{SiO}}_{2}\cdot yH_{2}O+(3-x){\mathrm{Ca}(\mathrm{OH})}_{2}$$

(2) $$2\mathrm{CaO}\cdot {\mathrm{SiO}}_{2}+xH_{2}O\to x\mathrm{CaO}\cdot {\mathrm{SiO}}_{2}\cdot yH_{2}O+(2-x){\mathrm{Ca}(\mathrm{OH})}_{2}$$

(3) $${\mathrm{Ca}(\mathrm{OH})}_{2}\to {\mathrm{Ca}}^{2+}+2{\mathrm{OH}}^{-}$$

(4) $${\mathrm{CaCl}}_{2}+{\mathrm{Ca}(\mathrm{OH})}_{2}+H_{2}O\to {\mathrm{CaCl}}_{2}\cdot {\mathrm{Ca}(\mathrm{OH})}_{2}\cdot H_{2}O$$

(5) $${\mathrm{CaCl}}_{2}+3{\mathrm{Ca}(\mathrm{OH})}_{2}+12H_{2}O\to {\mathrm{CaCl}}_{2}\cdot 3{\mathrm{Ca}(\mathrm{OH})}_{2}\cdot 12H_{2}O$$

#### 2.2.2. Hydration Heats of SWCMs

**Figure 3:**
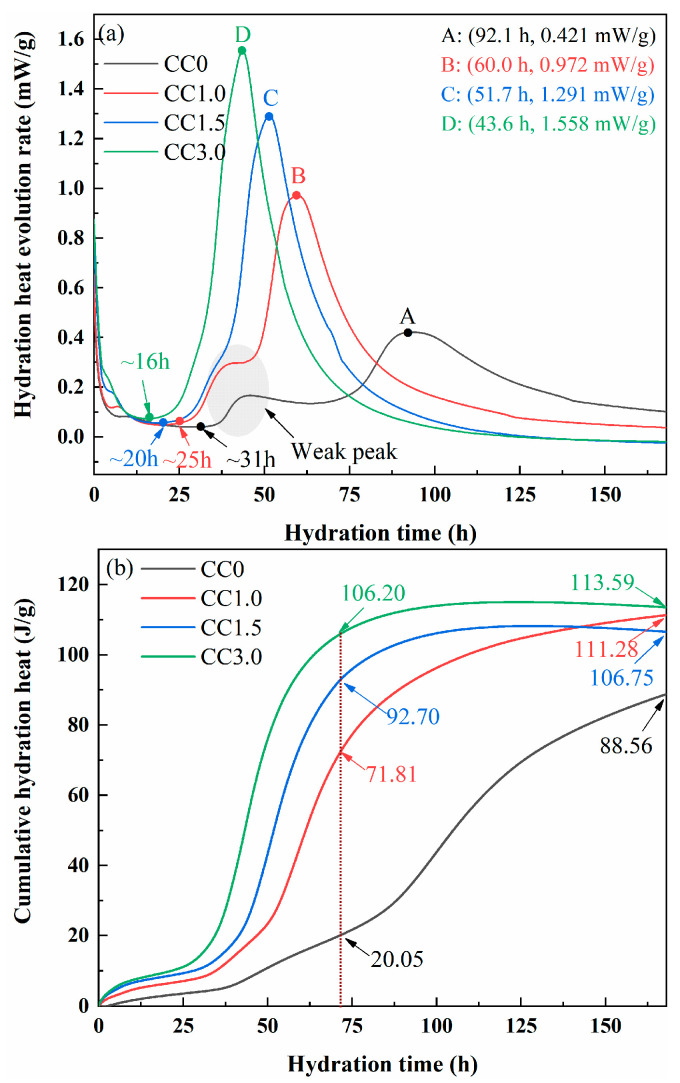
(**a**) Hydration heat flow curves and (**b**) cumulative heat curves of SWCMs with different CaCl_2_ contents.

Hydration heat flow curves of samples CC0, CC1.0, CC1.5, and CC3.0 are given in Figure 3a. For the control sample CC0 without adding CaCl_2_, the induction period persisted for as long as 31 h, while the duration of this period for samples CC1.0, CC1.5, and CC3.0 was shortened to 25 h, 21 h, and 16 h, respectively. This result indicates that CaCl_2_ accelerates the hydration reaction process of SWCMs. After the induction period, the hydration process of SWCMs enters the acceleration and deceleration period, in which large amounts of hydration products are generated and the microstructure gradually becomes denser [28,29]. Two distinct heat release peaks can be observed from the hydration heat flow curve of CC0. The first weak peak was mainly attributed to the rapid hydration of cement and steel slag. During the initial hydration stages, the C_3_S and C_2_S phases in the cement and steel slag could directly react with free water to form C-S-H gels and Ca(OH)_2_ (see Equations (1) and (2)) [30,31]. In addition, this peak may involve the rapid formation of ettringite (AFt) crystals via the reactions between mayenite in the steel slag or tetracalcium aluminoferrite in the cement and SO_4_^2−^ dissolved from desulfurization gypsum [32,33]. As Ca(OH)_2_ was continuously generated, the alkalinity of the pore solution increased gradually until the requirement for slag hydration was satisfied. In this case, the potential hydration reactivity of slag was activated, resulting in the second peak in the hydration heat flow curve. The exothermic rate of sample CC0 was relatively low, and its peak value was only 0.421 mW/g (at 92.1 h), indicating a slow hydration reaction. This should be the main reason for the low early strength of sample CC0. The exothermic rate of SWCMs was significantly accelerated with adding CaCl_2_, and the peak exothermic rate for samples CC1.0, CC1.5, and CC3.0 was 0.972, 1.291, and 1.558 mW/g, respectively, corresponding to the increase of 130.9%, 206.7%, and 270.0% compared to sample CC0, with an extremely significant enhancement effect. This result suggests that CaCl_2_ as an accelerator accelerates the hydration process of raw materials and prompts the production of hydration reactants efficiently. This is exactly how the compressive strength increases. Apart from these, only one exothermic peak was observed in the samples CC1.5 and CC3.0, possibly because the hydration rate of SWCMs was fast and the hydration reactions of raw materials such as cement, steel slag, and slag occurred almost simultaneously under the high CaCl_2_ contents.

The cumulative heat curves of samples CC0, CC1.0, CC1.5, and CC3.0 are shown in Figure 3b. There are significant differences in the cumulative hydration heat among the hydration samples with different CaCl_2_ contents during the initial hydration stages. The cumulative hydration heats of samples CC1.0, CC1.5, and CC3.0 are 71.81, 92.70, and 106.20 J/g at 3 days, respectively, with increases of 258.2%, 362.3%, and 429.7% compared to sample CC0. Increasing cumulative hydration heats of samples with CaCl_2_ indicates that CaCl_2_ plays a positive role in the reactants’ production, and the higher the CaCl_2_ content added is, the more hydration products are generated. As the hydration reactions proceeded, the difference in cumulative heat release among the samples decreased. At 7 days, the cumulative hydration heats of samples CC1.0, CC1.5, and CC3.0 are 111.28, 106.75, and 113.59 J/g, respectively, which only increase by 25.7%, 20.5%, and 28.3% compared to sample CC0. This phenomenon is possibly attributed to the hindering effect of the layer of hydration products. As reported in previous studies [28,34], with ongoing hydration reactions, the hydration products will precipitate on the surface of the raw material particles and form a dense hydration layer, which hinders further dissolution and hydration of raw material. At this time, the rate of reactant generation decreases, and the hydration process becomes stable thereafter.

#### 2.2.3. Hydration Products of SWCMs

##### XRD Analysis

**Figure 4:**
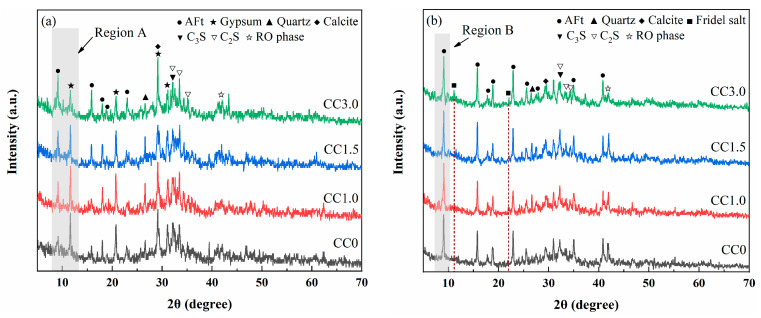

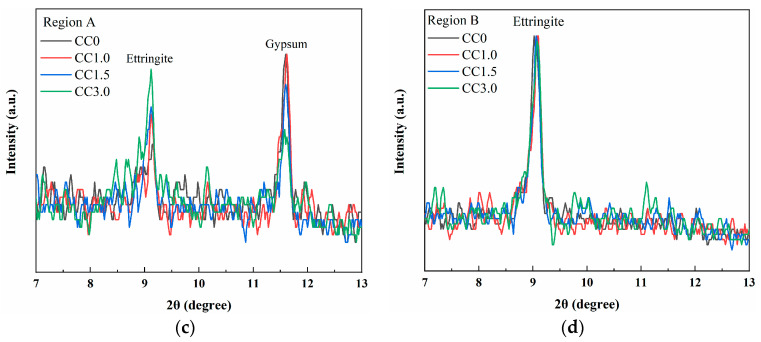
XRD patterns of (**a**) 3-day and (**b**) 28-day pastes under different CaCl_2_ contents; (**c**,**d**) enlarged views of the diffraction peaks for ettringite and gypsum between 7° and 13° (2θ).

XRD patterns of pastes with different CaCl_2_ contents are shown in Figure 4. Ettringite was identified as the main crystal product (AFt, 3CaO·Al_2_O_3_·3CaSO_4_·32H_2_O) in all hydrated samples. This reactant was typically generated in cementitious materials in the presence of SO_4_^2−^ sources, as shown in Equation (6), and it had been reported to substantially contribute to the early strength [35,36]. In addition to ettringite, a diffraction peak of gypsum (CaSO_4_·2H_2_O) was observed in hydrated samples after curing for 3 days, indicating that there was some unreacted desulfurization gypsum remaining in the samples during the early hydration stages. From the enlarged image of the strongest diffraction peaks of ettringite and gypsum (Region A), it can be clearly observed that the peak intensity of the ettringite increased continuously with the increase in CaCl_2_ content, while the peak intensity of the gypsum showed the opposite trend with CaCl_2_ content. These findings suggest that CaCl_2_ can facilitate the reaction of gypsum to produce ettringite, and this may be an important reason for the increase in the early strength of samples. The prompting effect of CaCl_2_ on ettringite production had been reported by Traetteberg et al. [37]. It was found that adding CaCl_2_ as an additive accelerated the dissolution of gypsum and prompted the formation of ettringite in the tricalcium aluminate–gypsum-based cementitious material.

(6) $${12\mathrm{Ca}}^{2+}+{{2\mathrm{Al}(\mathrm{OH})}_{4}}^{-}+{{3\mathrm{SO}}_{4}}^{2-}+{4\mathrm{OH}}^{-}+26H_{2}O\to \;3\mathrm{CaO}\cdot {\mathrm{Al}}_{2}O_{3}\cdot {3\mathrm{CaSO}}_{4}\cdot {32H}_{2}O$$

As the hydration age extended to 28 days, the diffraction peak of gypsum disappeared in all samples, and the diffraction peak intensity of ettringite significantly increased compared to that in the 3-day samples, suggesting that the gypsum was fully consumed during the production process of ettringite. As can be observed from the magnified image of the strongest diffraction peak of ettringite (Region B), the peak intensity of ettringite is essentially the same in samples with different CaCl_2_ contents. This may be because the amount of desulfurization gypsum in each sample is consistent, which determines that there is no significant difference in the ettringite production when the desulfurization gypsum is completely consumed at 28 days. In addition, the diffraction peaks 2θ = 11.10° and 22.05° were found in the samples containing CaCl_2_, which was attributed to the Cl-containing phase. As shown in Equation (7), Cl^−^ can react with Ca^2+^, Al^3+^, and OH^−^ to form Friedel salt (3CaO·Al_2_O_3_·CaCl_2_·10HO) [38,39]. The phase has good stability and is conducive to forming a harder skeleton during the hardening process and improving the macroscopic strength of the sample. It was worth noting that the diffraction peak of Friedel salt was not observed in the 3-day sample, mainly because its diffraction peak overlapped with the strong diffraction peaks of gypsum.

(7) $${{\mathrm{AlO}}_{4}}^{5-}+{\mathrm{CaCl}}_{2}+3\mathrm{CaO}+10H_{2}O\to 3\mathrm{CaO}\cdot {\mathrm{CaCl}}_{2}\cdot {\mathrm{Al}}_{2}O_{3}\cdot 10H_{2}O$$

It is important to note that the C-S-H gel, as the most dominant hydration product of CaO-rich cementitious materials, cannot be identified in XRD patterns due to its amorphous structure [40,41,42]. In the SWCM system, a portion of C-S-H gel is derived from the hydration of C_3_S and C_2_S in cement and steel slag, as shown in reaction Equations (1) and (2). In addition, another portion of C-S-H gel is associated with slag hydration. Si(OH)_4_ and Al(OH)_3_^−^ monomers can be dissolved from slag under an alkaline condition, and Si(OH)_4_ further reacts with OH^−^ to form [OSi(OH)_3_]^−^, [(OH)_2_SiO_2_]^2−^, and [(OH)SiO_3_]^3−^ oligomers, as shown in reaction Equations (8)–(10) [43,44]. Subsequently, the long aluminosilicate chains are formed by the polycondensation reactions between these oligomers via the attraction between hydroxyl groups, followed by combining with Ca^2+^ in the pore solution via physical electrostatic interactions to produce the final gel reactant [45,46].

(8) $${\mathrm{Si}(\mathrm{OH})}_{4}+{\mathrm{OH}}^{-}\to {{\left[\mathrm{OSi}(\mathrm{OH}\right)}_{3}]}^{-}+H_{2}O$$

(9) $${{\left[\mathrm{OSi}(\mathrm{OH}\right)}_{3}]}^{-}+{\mathrm{OH}}^{-}\to {{\left[(\mathrm{OH}\right)}_{2}{\mathrm{SiO}}_{2}]}^{2-}+H_{2}O$$

(10) $${{\left[(\mathrm{OH}\right)}_{2}{\mathrm{SiO}}_{2}]}^{2-}+{\mathrm{OH}}^{-}\to {{[(\mathrm{OH})\mathrm{SiO}}_{3}]}^{3-}+H_{2}O$$

##### TG-DTG Analysis

**Figure 5:**
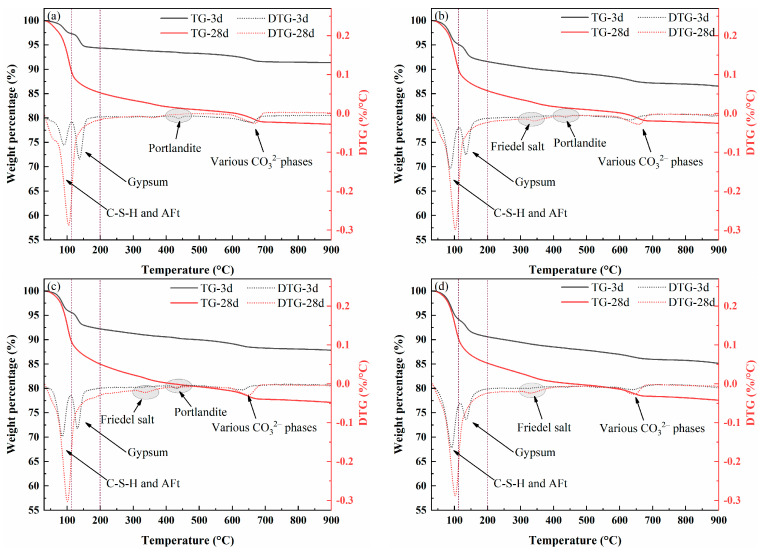
TG-DTG curves of pastes (**a**) CC0, (**b**) CC1.0, (**c**) CC1.5, and (**d**) CC3.0.To analyze the effect of CaCl_2_ content on the generation of various hydration products more intuitively, the weight loss of each hydration product in the hydrated sample was calculated based on the specific decomposition temperature range of each reactant, as shown in Table 1. The weight loss of sample CC0 without CaCl_2_ was only 3.04%, indicating that there was a small amount of C-S-H gel and ettringite. The weight loss of reactants increases with the increase in CaCl_2_ content, and the weight losses of samples CC1.0, CC1.5, and CC3.0 were 4.67%, 5.17%, and 6.21%, respectively. This result revealed that the addition of CaCl_2_ promoted the generation of hydration products. This is the reason why the sample strength increases with CaCl_2_ content. Contrary to the trend of weight loss of reactants, the weight loss of gypsum gradually decreased with CaCl_2_ content, indicating that CaCl_2_ accelerated the consumption of gypsum. This trend is consistent with the analysis results of XRD. In addition, the weight loss of Ca(OH)_2_ showed a constant decreasing trend with CaCl_2_ content. Specifically, the weight loss of Ca(OH)_2_ in samples CC0, CC1.0, CC15, and CC3.0 was 0.38%, 0.32%, 0.23%, and 0%, respectively. This result well explains the continuously decreasing trend of the pH of the pore solution with CaCl_2_ content.

To further analyze the effect of CaCl_2_ on the generation of hydration products, thermogravimetric analysis was performed. TG-DTG curves of CC0, CC1.0, CC1.5, and CC3.0 samples are presented in Figure 5. The weight-loss peak of the hydration sample mainly occurs between 30 °C and 200 °C. The weight-loss peak of 3-day samples between 30 and 200 °C consists of two parts: the weight-loss peak between 120 and 200 °C (the area between dashed lines) corresponds to the dehydration of C-S-H gel and ettringite, and the peak between 120 and 200 °C corresponds to the removal of crystal water in CaSO_4_·2H_2_O. As the hydration reactions proceeded, the gypsum was completely consumed, and the weight-loss peak of CaSO_4_·2H_2_O disappeared. As a result, the weight-loss peak between 30 and 200 °C in 28-day samples is mainly caused by the dehydration of C-S-H gel and ettringite. The decomposition peak of Friedel salt between 300 and 400 °C was observed in the CaCl_2_-doped samples [38], in agreement with XRD analyses. The weight-loss peak between 400 °C and 450 °C in the sample corresponds to the dehydroxylation of Ca(OH)_2_, while the weight-loss peak between 600 °C and 700 °C is attributed to the decomposition of the carbonate phases generated by the carbonization of the sample.

After curing for 28 days, the weight-loss peaks of gypsum and Ca(OH)_2_ disappeared. The weight loss of C-S-H gel and ettringite was significantly increased compared with the initial hydration period. The weight loss of hydration products in samples CC0, CC1.0, CC15, and CC3.0 is 10.15%, 11.23%, 11.58% and 10.78%, respectively. Different from the initial stage of hydration, the weight loss of reactants showed a decreasing trend when the CaCl_2_ content exceeded 1.5%. The analysis results of XRD suggest that the amount of ettringite generated in 28-day samples with different CaCl_2_ contents is approximately the same. Therefore, it is reasonable to think that the difference in weight loss of hydration products in 28-day samples is mainly caused by the C-S-H gel. For a given range, the addition of CaCl_2_ reduced the concentration of Ca(OH)_2_ in the pore solution, in turn promoting the hydration reaction of C_2_S and C_3_S in cement and steel slag and further increasing the production of C-S-H gel. Besides this, CaCl_2_ may accelerate the hydration of slag. For CaO or Ca(OH)_2_-activated slag cementitious materials, Cl^−^ readily reacts with Ca^2+^ and Al^3+^ dissolved from slag to generate a stable Friedel salt phase, reducing the saturation of active ions and breaking the hydration equilibrium of slag, which accelerates the further hydration of slag and produces larger quantities of C-S-H gel [47,48]. However, the pH of the pore solution of the hydration sample decreases significantly when the CaCl_2_ content is too high. In such a case, the dissolution and hydration of slag are instead inhibited, which is not conducive to the C-S-H gel formation.

##### SEM-EDS Analysis

**Figure 6:**
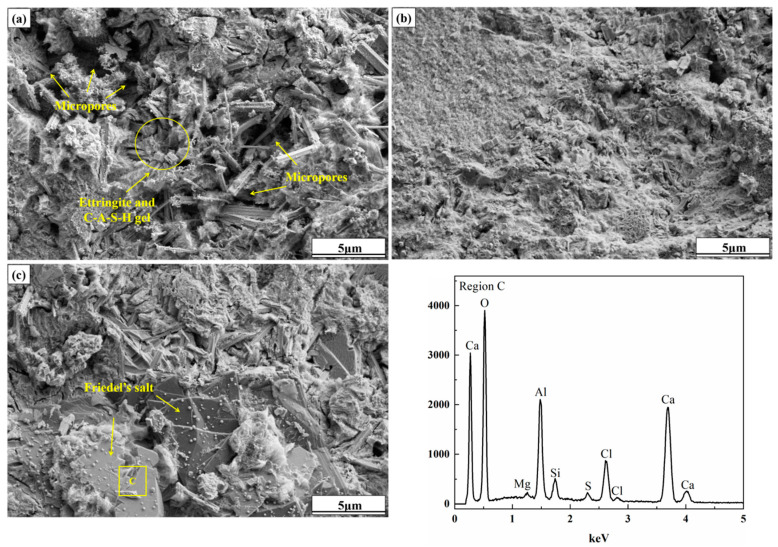
SEM images of samples (**a**) CC0, (**b**) CC1.5, and (**c**) CC3.0 at 28 days.

Figure 6 shows SEM images of samples CC0, CC15, and CC3.0 after 28 days. For sample CC0, amorphous C-S-H gel covered the surface of raw material particles to connect them together, and fibrous ettringite interspersed in the gaps between particles, forming a compact matrix. However, some micropores can still be observed in the sample. In comparison, larger quantities of C-S-H gel were generated in sample CC1.5 because of the prompting effect of CaCl_2_ on the gel formation. Precursor particles were completely wrapped by massive gel reactants, and the number of micropores between particles decreased clearly, as did the size, showing a more compact and homogeneous microstructure. The improvement of the microstructure of the sample has made a substantial contribution to the enhancement of the macroscopic strength. As the CaCl_2_ content further increased to 3.0%, in addition to amorphous C-S-H gel and fibrous ettringite, several lamellar products were found in sample CC3.0, which were stacked together and embedded in the sample. The EDS analysis indicates that the sheet-like product is rich in elements such as O, Ca, A1, and Cl, in line with the chemical composition of Friedel salts (3CaO·Al_2_O_3_·CaCl_2_·10HO). This phase with a stable structure can act as a skeleton to enhance the sample strength effectively [49].

### 2.3. Microstructural Analysis of Cemented Fine Tailings Backfill

#### 2.3.1. Microscopic Morphology Analysis

**Figure 7:**
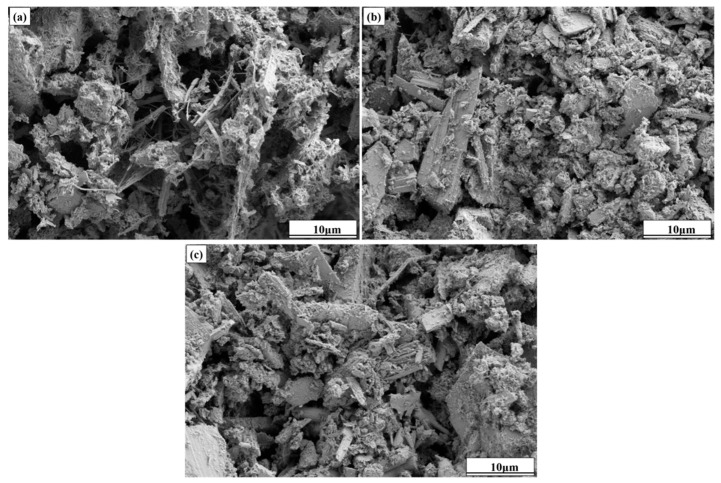
SEM images of cemented fine tailings backfills (**a**) CC0, (**b**) CC1.5, and (**c**) CC3.0 at 28 days.

The microscopic morphology of cemented fine tailings backfills at a binder-to-tailings ratio of 1:8 after curing for 28 days is shown in Figure 7. In sample CC0, the C-S-H gel with a network structure covered the surface of the tailing particles, and the fibrous ettringite was distributed in the void space between these particles. Tailings particles were loosely stacked together, and a large number of pores could still be observed in sample CC0, with a relatively rough microstructure. In the SEM image of sample CC1.5, much higher amounts of gel reactants can be observed. C-S-H gel played binder-like roles in covering the surface of tailings particles and filling the spaces between them; thereby, these tailings particles were tightly gathered together to form a uniform and dense microstructure. The improvement of microstructure is an important reason for the increase in sample strength. The addition of 3.0 wt.% of CaCl_2_ did not significantly improve the microstructure of sample CC3.0 but increased the number of voids. Based on the analysis results in Section 2.2, this phenomenon may be related to the reduction in C-S-H gel production at an excessive amount of CaCl_2_. The decrease in the compactness of the microstructure led to the degraded compressive strength.

#### 2.3.2. Pore Structure Analysis

**Figure 8:**
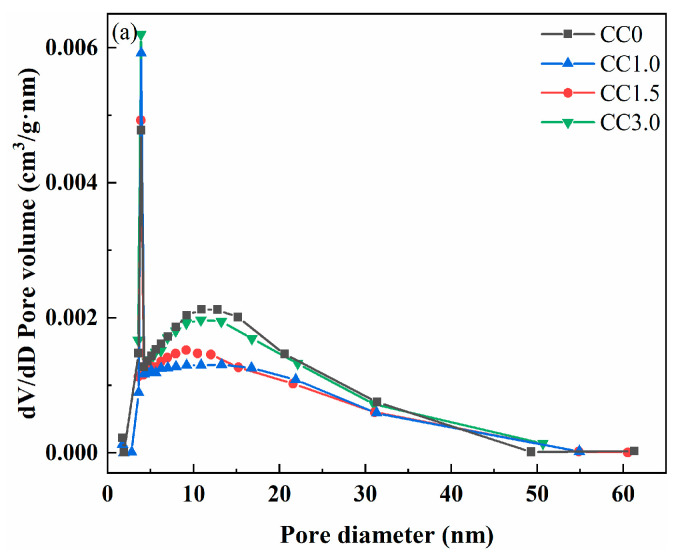

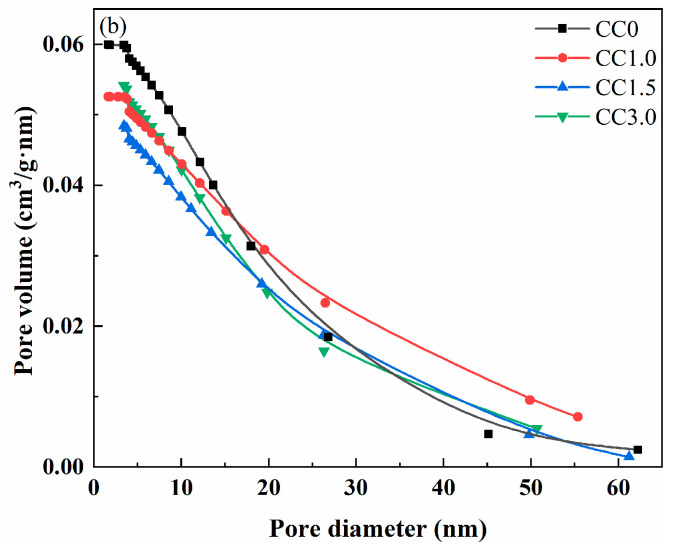
(**a**) Pore size distribution and (**b**) pore volume of 28-day cemented fine tailings backfills.

The pore size distribution and pore volume of cemented fine tailings backfills at a binder-to-tailings ratio of 1:8 after curing for 28 days are shown in Figure 8, and the pore structure parameters of these samples are presented in Table 2. Both pore volume and average pore diameter of samples showed a trend of first decreasing and then increasing with continued addition of CaCl_2_, which was consistent with the development trend of the sample strength. For sample CC0 without CaCl_2_, the pore volume and average pore diameter were 0.060 cm^3^/g and 14.160 nm, respectively. By contrast, the pore volume and average pore size of sample CC1.5 decreased to 0.048 cm^3^/g and 12.499 nm, respectively. In the presence of CaCl_2_ as an accelerator, larger amounts of hydration products were generated and endowed the cementitious material with better cementing performance for fine tailings, resulting in the improvement of the pore structure. As CaCl_2_ content further increased to 3.0 wt.%, the pore volume and average pore diameter of sample CC3.0 instead increased to 0.054 cm^3^/g and 13.246 nm, respectively. This is because the addition of excessive CaCl_2_ is unfavorable to the formation of reticular C-S-H gel resulting from the reduced pH of the pore solution. As a result, the cementing performance of cementitious materials for fine tailings is somewhat attenuated; in this case, the pore volume and average pore diameter of the sample are increased.

## 3. Experimental Materials and Methods

### 3.1. Characterization of Experimental Materials

**Figure 9:**
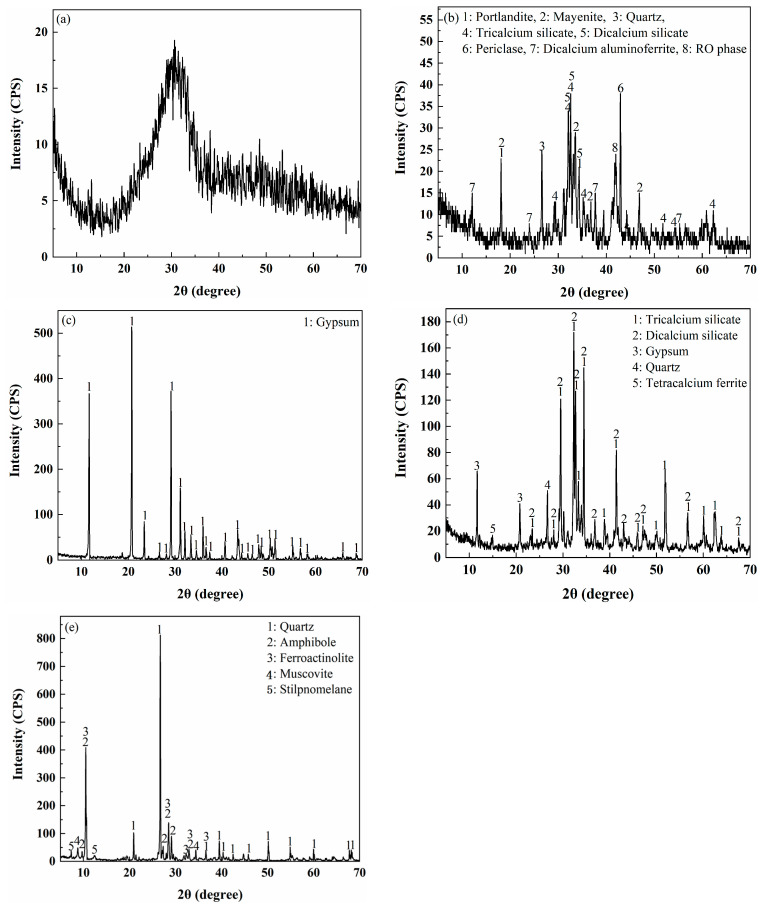
XRD patterns of (**a**) slag, (**b**) SS, (**c**) DG, (**d**) OPC, and (**e**) MTs.

Slag (produced from pig-iron production), steel slag (discharged from the steelmaking process), desulfurization gypsum (a byproduct generated during flue gas desulfurization in coal-fired power plants), and OPC were obtained from Shenfei Construction Material Co., Ltd., Qingdao, China. The 3-day and 28-day strengths of the OPC mortar are 17.41 and 44.35 MPa, respectively. Mine tailings (MTs, discharged from the mineral processing of iron ore) were provided by Fuquan Iron Minel Co., Ltd., Jining, China. The proportion of fine particles (less than 20 μm) of MTs is ~68.72%, and they are classified as fine tailings [5,9]. The chemical compositions of the raw materials were analyzed by an X-ray fluorescence instrument, and the results are shown in Table 3. The XRD patterns of raw materials are presented in Figure 9. Anhydrous calcium chloride (CaCl_2_, AR, ≥98%) was used as an accelerator.

### 3.2. Preparation and Characterization of Cemented Tailings Backfills

SWCMs from 42.5 wt.% slag, 27.5 wt.% SS, 15.0 wt.% DG, and 15.0 wt.% OPC were used as binders for preparing the cemented tailings backfills. During this process, CaCl_2_ was used as an accelerator, and its proportion varied from 0 to 3.0 wt.%, relative to the total mass of SWCMs. With a constant concentration of tailings slurry of 55%, the tailings slurry, binder, and accelerator were mixed for 5 min at the required binder-to-tailings ratio by mass (1:4, 1:6, and 1:8), and then the homogeneous cemented tailings backfill was poured into a stainless steel mold with a size of 40 mm × 40 mm × 160 mm. The mold was vibrated 60 times on a cement mortar vibrating platform and cured at 20 °C and 90% relative humidity for the required age. According to the CaCl_2_ proportion, cemented tailings backfills were labeled as CC0–CC3.0.

The compressive strengths of cemented tailings backfills were measured using an electronic universal testing machine (ZwickZ050, Ulm, Germany). Each strength test was performed in triplicate, and the test results were the average of the three tests.

After the strength test, the fractured samples were immersed in absolute ethanol for 72 h and then dried under vacuum at 50 °C for 72 h. One part of the dried samples was processed for microstructural morphology analysis using scanning electron microscopy (SEM, Hitachi SU8020, Tokyo, Japan) with an accelerating voltage of 5 kV. A fraction of bulk samples with a size of ~5 mm was processed for pore structure analysis on a surface area and porosity analyzer (ASAP 2460, Macrometrics, Norcross, GA, USA).

### 3.3. Preparation and Characterization of Pastes

According to the standard [50], SWCMs, CaCl_2_, and water were mixed at a water-to-solid (by mass) of 0.5 for 5 min to obtain the homogeneous paste. The CaCl_2_ proportion varied from 0 to 3.0 wt.%, relative to the total mass of SWCMs. The fresh paste was poured into a plastic cup and cured at 20 °C and 90% relative humidity for the required age. After curing, the pastes were broken and immersed in absolute ethanol for 72 h, followed by drying under vacuum at 50 °C for 72 h. One part of the sheet dried pastes was selected for SEM analysis with an accelerating voltage of 5 kV, and one part of pastes was ground to below 74 μm for the X-ray diffraction (XRD) and thermogravimetric (TG) analyses. XRD patterns of pastes were obtained on an X-ray diffractometer (Rigaku SmartLab SE, Tokyo, Japan), with a scanning angle from 5 to 70° (2θ) and a speed of 10°/min. TG curves of pastes were recorded using a STA7300 Thermo gravimetric analyzer (Hitachi Ltd., Tokyo, Japan) from 30 °C to 900 °C with a heating rate of 15 °C/min under a nitrogen atmosphere.

After curing for 3, 7, and 28 days, the hydrated pastes were broken and ground to below 425 μm. After grinding, 10.00 g of pastes and 100.00 g of pure water were mixed and shaken for 6 h at 25 °C according to the standard [51]. A PHSJ-6L acidity meter was used to measure the pH of the supernatant.

Then, 3.00 g of powders of SWCMs and CaCl_2_ were mixed with 1.50 g of pure water in an ampoule bottle. After mixing, the ampoule bottle was sealed and placed in an isothermal calorimeter (TAM Air, Malvern, Orsay, France) to record the hydration heats of pastes within 7 days at 20 °C.

## 4. Conclusions

The addition of CaCl_2_ as an accelerator significantly prompts the hydration process of SWCMs, enhancing the cementing performance of SWCMs for fine tailings. The main conclusions are as follows:

(1) CaCl_2_ reacted readily with active ions (e.g., Ca^2+^, Al^3+^, and OH^−^) dissolved from the raw materials in the pore solution to form insoluble Cl-containing salts, such as Friedel salt (3CaO·Al_2_O_3_·CaCl_2_·10HO). These stable phases contributed to the formation of the harder skeleton of samples, which may be one of the reasons for the increase in the sample strength. More importantly, the rapid consumption of active ions associated with the formation of Cl-containing salts, in turn, accelerated the further dissolution of raw materials and prompted the formation of larger amounts of C-S-H gel and ettringite, thus enhancing the cementing performance of SWCMs for fine tailings.

(2) The hardening of cemented fine tailings backfills is tightly connected to the combined effect of C-S-H gel and ettringite. The amorphous C-S-H gel with a high specific surface covered the surface of tailings particles and gathered them together, and fibrous ettringite was interspersed in the gaps between tailings particles and enhanced the cohesive bonding force among them. As the hydration reactions of SWCMs proceeded, C-S-H gel and ettringite were continuously generated, the pore volume and average pore diameter of the cemented fine tailings backfills consistently decreased, and the microstructures of the cemented fine tailings backfills became increasingly dense, ultimately forming a hardened material.

(3) The optimal amount of CaCl_2_ was controlled at 1.5 wt.%, relative to the total mass of SWCMs. In this case, the hardening duration of cemented fine tailings backfills was shortened to 36 h, with a 50% decrease compared to that of the control sample without CaCl_2_. The 36 h and 28-day strengths of cemented fine tailings backfills could reach 0.21 and 1.08 MPa, respectively, even at a low binder-to-tailings ratio of 1:8. These strengths reach the requirement of common cemented tailings backfills. The rapid hardening of cemented fine tailings backfills has significant implications for accelerating ore mining speed, improving mining production capacity, and ensuring the safe environment of underground mining sites.

(4) In this study, the reinforcement effect of CaCl_2_ on the cementation performance of solid-waste-based cementitious materials for fine tailings was studied based on the analyses of the pH of the pore solution, hydration products, hydration heats, microstructural morphology, and pore structure of samples. The addition of CaCl_2_ can significantly accelerate the hardening speed and achieve the satisfactory compressive strength of the cemented fine tailings backfill. However, the environmental safety of cemented fine tailings backfills with solid-waste-based cementitious materials needs to be further investigated in future research, especially in relation to groundwater.

## Figures and Tables

**Table 1:** Weight loss of each hydration product of samples at different curing ages (%).

Hydration Age	Hydration Products	Weight Loss			
		CC0	CC1.0	CC1.5	CC3.0
3 days	C-S-H and AFt	3.04	4.67	5.17	6.21
	CaSO_4_·2H_2_O	3.24	3.19	2.81	2.51
	Ca(OH)_2_	0.38	0.32	0.23	0
	Carbonate	1.60	1.58	1.68	1.45
28 days	C-S-H and AFt	10.15	12.23	12.58	11.78
	CaSO_4_·2H_2_O	0	0	0	0
	Friedel salt	0	0.48	0.75	0.89
	Ca(OH)_2_	0	0	0	0
	Carbonate	2.44	2.37	2.29	2.26

**Table 2:** Pore structure parameters of cemented fine tailings backfills with different CaCl_2_ contents.

Sample	CC0	CC1.0	CC1.5	CC3.0
Pore volume (cm^3^/g)	0.060	0.052	0.048	0.054
Average pore diameter (nm)	14.160	13.559	12.499	13.246

**Table 3:** Chemical compositions of raw materials (wt.%).

Components	Slag	SS	DG	OPC	MTs
SiO_2_	33.16	16.70	0.82	25.46	61.95
Al_2_O_3_	16.31	5.94	0.97	11.98	6.34
CaO	38.86	40.38	41.81	49.60	4.05
Fe_2_O_3_	0.36	19.66	0.22	3.72	23.18
MgO	8.37	8.04	0.77	3.61	2.85
MnO_2_	0.67	3.01	-	-	-
Na_2_O	0.71	-	0.25	-	0.61
K_2_O	-	-	-	0.91	-
SO_3_	1.25	0.75	54.17	3.46	1.02

## Data Availability

The original contributions presented in the study are included in the article, and further inquiries can be directed to the corresponding author.
